# SSAW: A new sequence similarity analysis method based on the stationary discrete wavelet transform

**DOI:** 10.1186/s12859-018-2155-9

**Published:** 2018-05-02

**Authors:** Jie Lin, Jing Wei, Donald Adjeroh, Bing-Hua Jiang, Yue Jiang

**Affiliations:** 1 College of Mathematics and Informatics, Fujian Normal University, Fuzhou, 350108 People’s Republic of China; 2 Lane Department of Computer Science and Electrical Engineering, West Virginia University, Morgantown, 26506 WV USA; 3 Department of Pathology, University of Iowa, Iowa city, 52242 Iowa USA

**Keywords:** *k*-mers, Wavelet transform, Complex numbers, Sequence similarity, Frequency domain

## Abstract

**Background:**

Alignment-free sequence similarity analysis methods often lead to significant savings in computational time over alignment-based counterparts.

**Results:**

A new alignment-free sequence similarity analysis method, called SSAW is proposed. SSAW stands for Sequence Similarity Analysis using the Stationary Discrete Wavelet Transform (SDWT). It extracts *k*-mers from a sequence, then maps each *k*-mer to a complex number field. Then, the series of complex numbers formed are transformed into feature vectors using the stationary discrete wavelet transform. After these steps, the original sequence is turned into a feature vector with numeric values, which can then be used for clustering and/or classification.

**Conclusions:**

Using two different types of applications, namely, clustering and classification, we compared SSAW against the the-state-of-the-art alignment free sequence analysis methods. SSAW demonstrates competitive or superior performance in terms of standard indicators, such as accuracy, F-score, precision, and recall. The running time was significantly better in most cases. These make SSAW a suitable method for sequence analysis, especially, given the rapidly increasing volumes of sequence data required by most modern applications.

## Background

Efficient and accurate similarity analysis for a large number of sequences is a challenging problem in computational biology [1, 2]. Alignment-based and alignment-free sequence similarity analysis are the two primary approaches to this problem. However, the huge computational time requirement of the traditional alignment-based methods is a major bottleneck [3]. Alignment-free methods have continued to grow in popularity, given their high time efficiency and competitive performance with respect to accuracy [3–5].

Over the years, alignment-free methods have been used on various sequence analysis problems in biology and medicine, including DNA sequences [6–8], RNA sequences [9], protein sequences [10, 11], as well as in detection of single nucleotide variants in genomes [12], cancer mutations [13], analysis of genetic gene transfer [14, 15], and even in clinical practice [16]. Although initially developed for problems in computational biology [17–22], alignment-free methods have found significant applications in many other application areas, e.g., computer science [1, 2], graphics [23], and forensic science [24].

Alignment-free approaches are broadly divided into two groups [3]: word-based methods and information theory based methods. Word-based methods commonly divide sequences into words(also called *k*-mers, *k*-tuples, or *k*-strings) in order to compare their similarity (/dissimilarity) [25]. Information theory based methods usually evaluate the informational content of full sequences [26–29]. According to Bonhamcarter et al. [25],the word-based methods can be further divided into five categories, namely, base-base correlations (BBC), feature frequency profiles (FFPs), compositional vectors(CVs), string composition methods, and the *D*_2_-statistic family.

Our proposed SSAW method is more closely related to the feature frequency profiles under the word-based methods [25]. Bonhamcarter et al. [25] surveyed 14 different alignment-free word-based methods [27, 29–37]. Many new approaches continue to emerge [3, 38–41]. Among them, the Wavelet-based Feature Vector(WFV) model by Bao et al. [41] transformed DNA sequences into a numeric feature vector for further classification. Our work is inspired by this transformation.

The Fourier transform has been attempted to convert DNA sequences to different feature vectors and was reported to be efficient [42–45]. Although the Fourier transformation is able to clearly characterize a sequence in the frequency domain, it is not sensitive to the time domain. The wavelet transformation has been used to overcome this shortcoming [46, 47]. Haimovich et al. [48] studied DNA sequences of different functions, and found that the wavelet transform of the DNA walk constructed from the varied genome sequences (from short to long nucleotide sequences) provides an effective representation for sequence analysis. Nanni et al. [49] used wavelet trees to combine different features to improve classification performance.

The discrete and stationary wavelet transforms are popular approaches in signal analysis using wavelets [50]. Bao et al. [41] proposed Wavelet-based Feature Vector (WFV) model where DNA sequences were discretely transformed into digital sequences according to the rules of *A*=0, *C*=1, *G*=2, and *T*=3. The local frequency entropy of the sequence based on the location distribution and word frequency of the base is calculated. A feature vector with fixed length representing a DNA sequence is extracted by using the Discrete Wavelet Transformation (DWT). The stationary wavelet transformation is reported to be lossless [51] and provides a better performance in image transformation than the discrete counterpart [52, 53]. The major reason is that the Discrete Wavelet Transform (DWT) has a downsampling step which discards information in the process. Because the stationary discrete wavelet transform does not have a downsampling step, the length of the approximation coefficients are the same as the input signal after decomposition. Hence, the stationary wavelet transformation is used in this study.

Thus, the proposed SSAW (Sequence Similarily Analysis using the Stationary Discrete Wavelet Transform) model is based on the stationary wavelet transformation. The *k*-mers of different lengths are extracted from the sequences and transformed into a feature vector with complex numbers by mapping to an unit circle. This process reduces the dimensionality of the data and also improves the computation speed. The experimental results show the effectiveness of the SSAW approach, demonstrating improved accuracy and faster running time, when compared with WFV, and other recent approaches. Below, we provide a brief description on the stationary discrete wavelet transform.

### Stationary discrete wavelet transform

Given a function *x*(*t*), its continuous wavelet transformation, *CWT*(*x*) is obtained by applying a mother wavelet function $\psi ^{*}\big (\frac {t-b}{a}\big)$, as shown in Eq. 1:

1 $$ CWT_{x}(a,b)=\frac{1}{|\sqrt{a}|}\int^{\infty}_{-\infty}x(t)\psi^{*}\left(\frac{t-b}{a}\right)dt  $$

where, *CWT*_*x*_(*a*,*b*) is the wavelet transform for the signal x(t), *a* is the scale parameter, *b* is the translation distance, and $\psi ^{*} \big (\frac {t-b}{a}\big) $ is the mother wavelet function.

A common practive is to discretize the scale and translation parameters by the power series. Variables *a* and *b* can be respectively discretized as follows:

$$a=a_{0}^{j}, b=nb_{0}a_{0}^{j}; \text{where}\ j,n\in Z, a_{0},b_{0}\in Z, \text{and}\ a_{0}\neq 1. $$

In general, *a*_0_=2, and *b*_0_=1. Then the mother wavelet can be expressed as:

$$\psi_{j,n}(t)=2^{\frac {-j}{2}} \psi\left(2^{-j}t-n\right) $$

Thus, the corresponding discrete wavelet transform is given by:

2 $$ DWT_{x}(j,n)=2^{-\frac{j}{2}}\int^{\infty}_{-\infty}x(t)\psi_{j,n}^{*}\left(\frac{t}{2^{j}}-n\right)dt  $$

where, *j* is the scale parameter, and *n* is the translation distance.

The wavelet transform has the ability to characterize the local characteristics of the signal in both the time domain and the frequency domain. It is a time-frequency localized analysis method which can change the time window and frequency domain window with multi-resolution analysis. The wavelet transform obtains the time information of the signal by translating the parent wavelet. The frequency characteristics of the signal are obtained by scaling the width of the parent wavelet.

With the discrete wavelet transform(DWT), each time the signal is decomposed, it is also downsampled. This means that the sampled signal has to be chosen from one of even signal or odd signals (and not both). That is, with one decomposition process, half of the data is lost. Therefore, with increasing DWT decomposition steps, the extracted signals will lose significant time-shifted information in the original sequence. The stationary wavelet transform (SWT) does not apply the downsampling process. Thus, it preserves the information in the original sequence better. The SWT decomposition method yields the approximation coefficients and the detail coefficients. The approximation coefficients preserves most of the information and reflects the transformation characteristics of the signal. The detail coefficients mainly preserves the local and noise characteristics of the signal, and can be discarded. In this work, only the approximation coefficients are used in representing the input sequence.

The proposed SSAW model uses a simple Haar mother wavelet to construct the feature vector. The Haar wavelet has a tightly supported orthogonal wavelet with short support length. The Haar wavelet function *ψ*_*H*_ is defined as follows:

3 $$ \psi_{H} (x)= \left\{ \begin{array}{rl} 1 & 0 \le x \le \frac{1}{2}\\ -1 & \frac{1}{2} < x \le 1\\ 0 & otherwise\\ \end{array} \right\}  $$

Different mother wavelets have different time-frequency characteristics. In the time-frequency analysis window, the smaller the width of the time domain window, the better the performance of the parent wavelet in time domain analysis. Similarly, the smaller the width of the frequency domain window, the better the performance of the parent wavelet in frequency domain analysis.

## Methods

### Detailed steps

There are four steps in our proposed SSAW method. First, *k*-mers are extracted from a sequence and their corresponding frequencies are counted and standardized/normalized. Second, each *k*-mer is transformed into a complex by mapping the *k*-mers to an unit circle. Third, the stationary wavelet transformation is performed on the resulting sequence of complex numbers. Finally, clustering and/or classification is applied as needed, depending on the specific application of interest.

#### Step 1: *k*-mer extraction and frequency standardization

Given a genetic sequence *S* of length *M*, *k*-mers are extracted from the sequence by passing a sliding window of length *k* (varied from 2 to *M*−1) over the sequence. There are *M*−*k*+1 total *k*-mers in a sequence with length *M*. And there are at most |*Σ*|^*k*^ individual *k*-mers for a sequence with |*Σ*| alphabets. For a fixed *k*, a unit circle is divided evenly into |*Σ*|^*k*^ parts. A DNA sequence consists of symbols from the alphabetic *Σ*={*A*,*C*,*G*,*T*}, then |*Σ*|=4. A protein sequence consists of symbols from a larger alphabet, *Σ* = {*A*, *C*, *D*,*E*,*F*,*G*,*H*,*I*,*K*,*L*,*M*,*N*,*P*,*Q*,*R*,*S*,*T*,*V*,*W*,*Y*}, with |*Σ*|=20.

Let *X*_*t*_ denote the frequency of the *t*-th *k*-mer in a sequence and let *S*_*t*_ represent the standardization of *X*_*t*_ by using *z*-score normalization, as shown in Eq. 4.

4 $$ S_{t}= \frac{X_{t}-\overline{X}}{sd}  $$

where $\overline {X}$ represents the mean frequency of a *k*-mer *X* occuring in all the sequences. The denominator *sd* denotes the standard deviation of the frequencies of the *k*-mer *X* in all the sequences.

Motivated by the work in [18, 54], we use the following recommended length for *k*, given by:

5 $$ k= \left\lceil \log_{|\Sigma|}\left(\sqrt{|S|}\right)\right\rceil=\left\lceil \frac{\log_{|\Sigma|}(|S|)}{2} \right\rceil  $$

where |*S*| is the average of a sequence length.

#### Step 2: Transform *k*-mers to complex numbers

For a sequence with symbols from an alphabet *Σ*, there are at most |*Σ*|^*k*^ unique *k*-mers. First, sort all *k*-mers alphabetically. Given a unit circle, we evenly distribute all the |*Σ*|^*k*^*k*-mers around the circumference of the unit circle, moving counterclockwise. A *k*-mer is transformed into a complex number as follows:

- The sine of the angle the *k*-mer resides in becomes the real part of a complex number;
- the cosine of the angle the *k*-mer resides in becomes the imaginary part of a complex number.

The angle of the *t*-th *k*-mer *φ*_*t*_ is given by:

6 $$ \varphi_{t}=\frac{360}{|\Sigma|^{K}}\times t  $$

where *t* denotes the position of the *t*-th *k*-mer in *Σ*^*k*^.

Thus, the complex number representation for the *t*-th *k*-mer will be given by : <*Real*_*t*_,*Imag*_*t*_>=<*sin*(*φ*_*t*_),*cos*(*φ*_*t*_)>, where *Real*_*t*_= sin(*φ*_*t*_) is the real part, and *Imag*_*t*_= cos(*φ*_*t*_) is the imaginary part.

#### Step 3: Stationary wavelet transformation

After a sequence is transformed into a series of complex numbers, the real and imaginary parts of the complex numbers are multiplied by the corresponding standardized frequency (*S*_*t*_) of *k*-mers from the first step. And then, the stationary wavelet transformation is performed. Given an original string *S*, let *CODE*_*S*_ denote the series of complex numbers which are the combination of the real part and the imaginary part based on the sequence of *k*-mers. We apply the Haar transformation on *CODE*_*S*_ as shown in Eq. 7.

7 $$ F(S)=HaarSDWT_{AC}\left(CODE_{S},L\right)   $$

where, *F*(*S*) denotes the feature vector representing sequence *S*, and *L* is the decomposition level. The function *HaarSDWT*_*AC*_() denotes the SDWT using the Haar mother wavelet, while retaining the AC coefficients. We use the package SWT2 [55] in MATLAB for this transformation. A feature vector *F*(*S*) is obtained after the transformation.

#### Step 4: Clustering/classification using the feature vectors.

After the above processing, a text sequence is transformed into a feature vector. These feature vectors can then be used in clustering and classification applications. For proof of concept, we applied a simple clustering technique(namely, the *k*-means clustering algorithm) on the feature vectors. Similarly, for classification, we applied simple classification approaches (namely, *k*-Nearest Neighbor approach, using just *k*=1). In the classification experiment, the 1-Nearest Neighbour (*1-NN*) classification algorithm is applied. Finally, the experimental results are evaluated.

#### A simple example

Here, we discuss a simple example. Given two DNA sequences, *S1:AACAA* and *S2:CCGCC*. Assume that the sliding window length *K* is 2. There are |*Σ*|^*K*^= 4^2^=16 unique *k*-mers. The unit circle will be divided into 16 parts in this case.

As shown in Table 1, all 16 *k*-mers are listed on the first line. The frequency of a *k*-mer (*X*_*t*_) for a sequence is counted respectively. Many *k*-mers have a zero frequency in this simple example. However, in real applications, this is seldom the case, since the sequences are generally much longer. Similarly, the the standard deviation *sd* in the denominator are rarely zero. See Eq. 4. For the purpose of this demonstration only, we assume a series of non-zero values for *sd* which are shown on the last row in the table. The similar assumption is applied to $\overline {X}$ which is listed on the second last line.

**Table 1:** Length 2 *k*-mers and associated standardized frequencies (Eq. 4)

	k-mers	AA	AC	AG	AT	CA	CC	CG	CT	GA	GC	GG	GT	TA	TC	TG	TT
S1	*X*_*t*_	2	1	0	0	1	0	0	0	0	0	0	0	0	0	0	0
	*S*_*t*_	0.07	-0.84	-0.17	-0.38	-0.76	-0.76	-0.55	-0.38	-0.09	-0.76	-0.42	-0.14	-0.09	-0.35	-0.18	-0.3
S2	*X*_*t*_	0	0	0	0	0	2	1	0	0	1	0	0	0	0	0	0
	*S*_*t*_	-0.41	-1.13	-0.17	-0.38	-1.02	-0.23	-0.29	-0.38	-0.09	-0.48	-0.42	-0.14	-0.09	-0.35	-0.18	-0.3
	$\overline {X}$	1.7	3.9	0.9	1.3	3.9	2.9	2.1	1.3	0.3	2.7	1.5	0.7	0.3	1.2	0.7	1.1
	sd	4.14	3.45	5.17	3.45	3.84	3.84	3.84	3.45	3.45	3.55	3.55	5.07	3.45	3.45	3.89	3.71

Then, Eq. 4 is applied to calculate the corresponding standard deviation (*S*_*t*_) of a *k*-mer. For example, for the first *k*-mer *AA* in sequence *S*1, the normalized value is $\frac {2-1.7}{4.14}=0.07$.

**Fig. 1:**
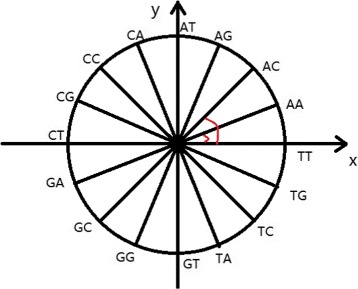
The distribution of 16 *k*-mers (AA, AC, …, TT) on the unit circle, moving counterclockwise

In the second step, the unit circle is divided into 16 equal parts. Since length of *k*-mer is assumed to be 2 here, there are |*Σ*|^*K*^= 4^2^=16 possible unique *k*-mers. These 16 *k*-mers are distributed on the unit circle in a counterclockwise manner, as shown in the Fig. 1.

Each *k*-mer has a corresponding radian measurement. For example, for the first *k*-mer *AA*, the radian is $\frac {360}{|\Sigma |^{K}}\times t=\frac {360}{4^{2}}\times 1$=22.5. We have *Real*_*t*_= sin(22.5)=0.38. The imaginary part of the complex number value is: *Imag*_*t*_= cos(22.5)=0.92. Hence, the corresponding *k*-mer *AA* in sequence *S*1 is represented as a complex number (0.38,0.92). Then, the standardized frequency *S*_*t*_ (0.07) from the first step is multiplized to this complex number (0.38,0.92), resulting in the pair (0.0266,0.0644).

After processing all the *k*-mers, a series of complex numbers starting with (0.0266,0.0644) are input into the third transformation step. After the third step (stationary wavelet transform), a feature vector will be obtained which can then be used for clustering and/or classification.

### Distance measurement

The similarity between feature vectors is measured using the Euclidean distance as follows.

8 $$ Eu_{d}(S_{1},S_{2})= \sqrt{\sum_{i=1}^{Vec}|F_{i}(S_{1})-F_{i}(S_{2})|^{2}}  $$

where *Vec* is the length of the feature vector, *F*(*S*_1_) and *F*(*S*_2_) denote feature vectors for sequences *S*_1_ and *S*_2_ respectively.

### The measurement of clustering assessment

The F-score is used to evaluate the clustering results. Let *C*_*i*_ represent the number of sequences in the family *i*; let *C*_*ij*_ represent the number of sequences belonging to cluster *j* in family *i*. *lb*(*j*) represents the family tag of cluster *j*, when clustering, the goal is to cluster a sequence in family *j* to be in cluster *lb*(*j*).

The sequences in family *i* are decided to belong to the cluster *j* by using dominating rule, the cluster that contains the largest number of sequences is selected to be *lb*(*j*), shown as in Eq. 9:

9 $$ lb(j)=argmax_{i=1}^{fm}\left(C_{ij}\right)  $$

where *fm* is the number of all possible families.

For a given family *i*, the respective values for precision, recall, and f-score are computed as follows:

10 $$ precision_{i}=\frac{\sum\limits_{lb(j)=i}C_{ij}}{\sum\limits_{lb(j)=i} \overline{ C_{j}}}  $$

where $\overline {C_{j}}$ represents the number of sequences in cluster *j*.

11 $$ recall_{i}=\frac{\sum\limits_{lb(j)=i}C_{ij}}{C_{i}}  $$

12 $$ F-score(i)=\frac{2 \times precision(i)\times recall(i) }{precision(i)+recall(i)}  $$

The *F*-score for all families can be calculated as:

13 $$ F-score=\sum_{i=1}^{fm}\frac{C_{i}}{C}F(i)  $$

where *C* is the total number of sequences in the dataset.

### The measurement of classification

We use the confusion matrix (see Table 2) to evaluate the classification performance. The confusion matrix is an *N*×*N* matrix, where *N* is the number of categories in the classification. We use the predicted and original categories to establish the confusion matrix.

**Table 2:** Confusion matrix

		Predicted class	
		Positive	Negative
Actual	Positive	True positives(TP)	False negatives(FN)
class	Negative	False positives(FP)	True negatives(TN)

Based on the above confusion matrix, the performance indicators are defined as follows.

- Accuracy = (TP+TN)/(TP+TN+FN+FP)
- Precision = TP/(TP+FP)
- Recall = TP/(TP+FN)
- F-score = 2*Precision*Recall/(Precision+Recall)

## Results

A new alignment-free sequence similarity analysis method, SSAW, is proposed. The performance of SSAW is compared against those of two methods, namely, WFV [41] and $K_{2}^{*}$ [18], which represent the current state-of-the-art. Compared with WFV and $K_{2}^{*}$, the SSAW method demonstrates competitive performance in clustering and classification, with respect to both effectiveness (accuracy), and efficiency (running time).

### Datasets

Three types of data are used in our experimental evaluation, namely, DNA sequences, protein sequences, and simulated next generation sequences. The DNA datasets are the same as those used in Bao et al.’s original paper [41]. The longest sequence has 8748 characters and the shortest sequence has 186 characters. The HOG datasets used contained 100, 200, 300 families, with a corresponding family size of 96, 113, and 93 DNA sequences, respectively.

The protein datasets were obtained from [41] too, which were randomly selected from HOGENOM by ourselves. They are also from HOG100, HOG200, and HOG300. The longest sequence has 2197 characters and the shortest sequence has 35 characters. The HOG protein datasets contained 100, 200, 300 families, with an average family size of 9, 10, 11, respectively. Both protein and DNA datasets were collected by the Institute of Biology and Chemistry of Proteins (IBCP), using PBIL (population-based incremental learning), and are available at: ftp://pbil.univ-lyon1.fr/pub/hogenom/release_06/.

The third data set is our simulated DNA next-generation sequences data with a total of 520 sequences of length 47 base pairs each. There are eight classes, each with 65 sequences. The original 8 sequences are randomly selected from a next-generation sequence data set (Illumina platform) for error correction [56]. During simulation, 8 sequences of length 47 with edit distance of 10 among them are randomly selected. These 8 sequences are regarded as the 8 data centroids. For each centroid, 64 sequences are generated with edit distance ≤ 4 from the centroid. These 8 centroids form our 8 cluster centers.

### Experimental design

The experiments were performed on a machine running Windows 7 Operating System (64 bit professional edition) with Intel Core i5-3470 (3.20 GHz) CPU and 8 GB RAM. The experiments were performed on the three types of data described, and their corresponding run times (in seconds) are also recorded. The reported execution times are averages, over several iterations.

Firstly, we check the validity of the proposed SSAW by comparing it against the standard edit distance [1, 2] and the global alignment identity score [5]. The edit distance between two strings is defined as the minimum number of edit operations required to transform one string into the other. The edit distance is the basic standard used to compare two strings [1, 2]. The Needleman-Wunsch alignment algorithm is the other golden standard in measuring sequence similarity [57]. They both have a quadratic time complexity with respect to the length of the strings which are computed using dynamic programming [58]. Thus, we randomly extract 100 sequences from the dataset for this validity check.

For clustering, *k*-means [59] in RGui is used. Proposed SSAW, WFV by Bao et al. [41], and $K_{2}^{*}$ by Lin et al. [18] are assessed by using F-score, precision, and recall. It is well known that, for *k*-means, the initial center is important. To diminish the influence of initial centers, the cluster center is selected randomly, and the experiment is repeated 200 times. The average value is then reported.

For classification experiment, we used the *1-NN* classification algorithm (*kNN* method with *k*=1). To reduce the random selection effect caused by dividing training sets and testing sets, the classification experiment is repeated 100 times and the average is reported. The stratification sampling is applied to select 80 percent of data for training, and the remaining 20 percent of data is used for testing.

The SSAW method has two parameters that need to be set, namely, the *k* value for *k*-mers, and the decomposition level *L* in the wavelet transformation stage. The value of *k* is determined by using Eq. 5, which is motivated by earlier work [18, 54]. After running all possible decomposition levels, our experiment showed that setting *L*=*k* is the most suitable in our applications. Hence, in SSAW, the recommended parameter values for *k* and *L* can be automatically determined by using Eq. 5. For WFV, the vector length is fixed at 32 which is recommended by the original authors [41].

#### Validity of the proposed SSAW

Two groups of correlation measures are calculated on two datasets, namely, DNA sequences, and protein sequence data. One is the correlation between edit distance and the respective results of the SSAW, WFV and $K_{2}^{*}$ methods. The other is the correlation between the global alignment identity score and the results of the SSAW, WFV, and $K_{2}^{*}$ methods. The global alignment identity score is calculated by using the Needleman-Wunsch algorithm [57]. 100 sequences are randomly selected from one cluster of DNA (and one family of protein sequences). Then, the edit distance, the global alignment score, and the results for SSAW, WFV and $K_{2}^{*}$ are calculated between pairs of sequences. Finally, the Pearson correlation coefficient is calculated between the edit distance and the respective results from the three methods. The same correlation is repeated using the global alignment identity score, rather than the edit distance. The correlation results are shown in Table 3.

**Table 3:** Correlations between edit distance (the global alignment identity score) and three methods

	DNA			Protein		
	SSAW	WFV	$K_{2}^{*}$	SSAW	WFV	$K_{2}^{*}$
*E* *d* *i* *t* *d* *i* *s* *t* *a* *n* *c* *e*	0.779	0.837	-0.67	0.852	0.861	-0.842
*I* *d* *e* *n* *t* *i* *t* *y* *s* *c* *o* *r* *e*	-0.741	-0.742	0.799	-0.841	-0.822	0.789

Looking at Table 3, one may wonder why some correlations is negative (positive). The reasons are as follows. The edit distance, SSAW and WFV are calculated by using distance measurements. Thus, the correlation between any two of these are positive. The global alignment identity score and $K_{2}^{*}$ calculate the similarity between sequences. Thus, the latter two are similar.

With the Pearson correlation coefficient, a value of 0 indicates no correlation; a value of 1 indicates positive correlation, while a value of −1 indicates negative correlation. For a comparison method, a value close to 1 or − 1 indicates its ability in measuring the similarity (/dissimilarity) between sequences. On the contrary, a value close to 0 shows an inability to measure the similarity (/dissimilarity) between the given sequences.

For Pearson correlation, we should consider their absolute values, rather than the direct correlation values. With this in mind, Table 3 shows that all the three methods are strongly correlated with the edit distance, and also with the global alignment identity score. This indicates that the three methods are all valid in measuring similarity between DNA (protein) sequences.

#### DNA data

Table 4 shows the experimental results for clustering DNA sequences using the three methods: SSAW, WFV, and $K_{2}^{*}$. The F-score is computed by combining values for precision and recall. Hence, for brevity, in the following, we will focus on F-score comparison. However, values for precision and recall will also be listed for reference purposes. From Table 4, we can find that SSAW has the best overall performance on all the three DNA data sets.

**Table 4:** Comparison of the clustering results on DNA dataset

DNA-Data	Model	F-score	Precision	Recall
HOG100	SSAW	0.6099	0.5953	0.6648
HOG100	WFV	0.5724	0.5569	0.6227
HOG100	$K_{2}^{*}$	0.5551	0.5112	0.6073
HOG200	SSAW	0.5982	0.5841	0.6508
HOG200	WFV	0.5635	0.5610	0.6214
HOG200	$K_{2}^{*}$	0.5788	0.5364	0.6285
HOG300	SSAW	0.5961	0.5869	0.6421
HOG300	WFV	0.5359	0.5434	0.5800
HOG300	$K_{2}^{*}$	0.5466	0.5081	0.5915

Table 5 shows the classification results generated from three models on DNA datasets. In the classification, one measurement, accuracy which is known as a comprehensive indicator, is evaluated. Studying Table 5, the first impression is that three models have similar values which are very close to each other. Using the accuracy measure, SSAW was slightly better on two datasets, HOG200 and HOG300, while $K_{2}^{*}$ was slightly better on HOG100. If we compare the F-score values, WFV was better on two datasets (HOG100 and HOG200), while SSAW was better on HOG300. Practically, we can say that these three models have similar performance, and that SSAW is competitive in this experiment.

**Table 5:** Comparison of the classification results on DNA datasets

DNA-Data	Model	Accuracy	F-score	Precision	Recall
HOG100	SSAW	0.9576	0.9315	0.9326	0.9305
HOG100	WFV	0.9574	0.9426	0.9475	0.9447
HOG100	$K_{2}^{*}$	0.9587	0.9335	0.9472	0.9202
HOG200	SSAW	0.9548	0.9256	0.9366	0.9149
HOG200	WFV	0.9544	0.9355	0.9430	0.9350
HOG200	$K_{2}^{*}$	0.9439	0.9320	0.9331	0.9309
HOG300	SSAW	0.9509	0.9311	0.9354	0.9268
HOG300	WFV	0.9402	0.9208	0.9286	0.9219
HOG300	$K_{2}^{*}$	0.9328	0.9255	0.9229	0.9282

Table 6 shows the corresponding running times for the three analysis methods in clustering and classification on DNA datasets. From Table 6, we can observe that for clustering, SSAW is the fastest method among the three. It runs much faster than WFV by as much as 3, 5, and 10 fold increases in speed. For classification of DNA sequences, WFV was the fastest method among these three methods. $K_{2}^{*}$ was faster than SSAW on two of the three data sets, but slower on one dataset.

**Table 6:** Running time for clustering and classification on DNA datasets. The fold improvement from a given method to the proposed SSAW approach is listed inside the parenthesis

DNA-Data	Model	Total	Total
		clustering time	classification time
HOG100	SSAW	19.8000	16.8159
HOG100	WFV	55.4619(3)	10.4614
HOG100	$K_{2}^{*}$	39.676(2)	11.3421
HOG200	SSAW	50.9515	51.5956
HOG200	WFV	238.5061(5)	26.8309
HOG200	$K_{2}^{*}$	104.327(2)	37.8473
HOG300	SSAW	63.9960	77.7017
HOG300	WFV	640.1409(10)	31.4625
HOG300	$K_{2}^{*}$	238.712(4)	94.8274

Combining the performance of these three models, we can note the following: (1) For clustering, the recommended method is SSAW, it not only has the best performance, but also has the fastest running time. (2) For classification, WFV would be the best choice which has the advantage of performance plus running time. However, SSAW demonstrated competitive performance, with respect to both accuracy and running time.

#### Protein data

Table 7 shows the clustering results on the protein sequence data. In all three data subsets, SSAW was the best.

**Table 7:** Comparison of the cluster results on protein data set

Protein-Data	Model	F-score	Precision	Recall
HOG100	SSAW	0.7651	0.7497	0.8001
HOG100	WFV	0.5874	0.5687	0.6382
HOG100	$K_{2}^{*}$	0.6604	0.642	0.6798
HOG200	SSAW	0.7746	0.7573	0.8103
HOG200	WFV	0.6410	0.6195	0.6913
HOG200	$K_{2}^{*}$	0.6435	0.5969	0.6979
HOG300	SSAW	0.7246	0.7088	0.7653
HOG300	WFV	0.5016	0.4826	0.5551
HOG300	$K_{2}^{*}$	0.6429	0.6111	0.6782

Table 8 shows the classification results generated using these three methods on protein data sets. Using accuracy for performance measurement, SSAW was the best on two data sets (HOG200 and HOG300), while $K_{2}^{*}$ performed best on the other data (HOG100). Using F-score, SSAW was best on HOG300 and $K_{2}^{*}$ was the best on the other two data subsets. Generally speaking, SSAW and $K_{2}^{*}$ were quite competitive in this experiment, while WFV generated inferior results. Table 9 shows the running time in clustering and classification on protein datasets. In all protein data sets and two applications, SSAW outperformed the other two methods overwhelmingly. WFV was the runner up, while $K_{2}^{*}$ could not compete on this dataset.

**Table 8:** Comparison of the classification results on protein data

Data	Model	Accuracy	F-score	Precision	Recall
HOG100	SSAW	0.8158	0.6274	0.6225	0.6644
HOG100	WFV	0.6741	0.5092	0.5012	0.5518
HOG100	$K_{2}^{*}$	0.8329	0.6540	0.6248	0.6861
HOG200	SSAW	0.8222	0.5626	0.5441	0.6174
HOG200	WFV	0.7051	0.4454	0.4359	0.4902
HOG200	$K_{2}^{*}$	0.8061	0.6279	0.5875	0.6743
HOG300	SSAW	0.8690	0.7345	0.7466	0.7642
HOG300	WFV	0.5685	0.3468	0.3551	0.3774
HOG300	$K_{2}^{*}$	0.8098	0.6308	0.5983	0.6670

**Table 9:** Running time for clustering and classification on protein datasets. The fold improvement from the a given method to the proposed SSAW is listed inside the parenthesis

Protein-data	Models	Total clustering	Total classification
		time	time
HOG100	SSAW	0.1638	0.1262
HOG100	WFV	5.5554(34)	0.4164(3)
HOG100	$K_{2}^{*}$	10.964(67)	1.3780(11)
HOG200	SSAW	0.3542	0.2738
HOG200	WFV	11.5037(32)	0.9362(3)
HOG200	$K_{2}^{*}$	49.016(138)	3.091(11)
HOG300	SSAW	0.6965	0.5077
HOG300	WFV	27.2514(39)	1.7460(3)
HOG300	$K_{2}^{*}$	126.984(182)	5.284(10)

Taken together, we can make a few notes on working with protein datasets: (1) SSAW generally has the best performance on clustering and classification using the protein datasets. (2) SSAW also has the fastest running time. (3) The $K_{2}^{*}$ was better than WFV on some cases, however, the required execution time was higher than that of WFV. (4) For WFV, the running time was second to SSAW, however, the accuracy was not as good. Overall, it appears that, when the alphabet size is increasing, the proposed SSAW method with its initial stage of mapping the *k*-mers to complex numbers based on the unit circle, produces superior results than the state-of-art.

#### Simulated data

Table 10 shows the results for clustering using the simulated datasets. We can see from Table 10, $K_{2}^{*}$ is the best one among these three methods. Comparing SSAW to WFV, WFV is slightly better than SSAW, although their performance numbers are quite close.

**Table 10:** Comparison of the clustering results on simulated dataset

Model	F-score	Precision	Recall
SSAW	0.8151	0.8085	0.8467
WFV	0.8211	0.8056	0.8587
$K_{2}^{*}$	0.8584	0.8750	0.8425

Table 11 compares the classification results of these three methods using the simulated data. WFV is the best one among the three. SSAW is second, performing better than $K_{2}^{*}$.

**Table 11:** Comparison of the classification results on simulated data

Model	Accuracy	F-score	Precision	Recall
SSAW	0.9789	0.9789	0.9804	0.9789
WFV	0.9992	0.9992	0.9993	0.9992
$K_{2}^{*}$	0.9607	0.9662	0.9696	0.9628

Table 12 describes the running times for these three methods on simulated data. Comparing three models, SSAW was the fastest. $K_{2}^{*}$ is the slowest in clustering. For clustering, the running times for $K_{2}^{*}$ and WFV were respectively, 18 and 15 times slower, than those of SSAW. In classification, the running time of $K_{2}^{*}$ and WFV were 11 and 2 times slower, respectively.

**Table 12:** Running time for three methods on clustering and classification using simulated data

Models	Total clustering	Total classification
	time	time
SSAW	0.0632	0.0810
WFV	0.9288(15)	0.9313(11)
$K_{2}^{*}$	1.123(18)	0.172(2)

Combining the performance and speed, we can note the following with respect to the simulated data: (1) SSAW and WFV can be recommended methods for clustering. The running time of $K_{2}^{*}$ is relatively high – 18 times more than SSAW and 1.2 times more than WFV. (2) For classification, SSAW is a good choice, with competitive performance and the fastest running time. WFV is the most accurate method, however, it has longer running time (11 times more than SSAW, and 5.4 times more than $K_{2}^{*}$).

Considering the three types of data used in the experiments, and the two applications considered, we can draw some overall conclusions. Table 13 summarizes the overall results of our analysis.

**Table 13:** Recommended methods for clustering and classification given three datasets. Model inside parentheses is competitive

Data	Cluster	Classification
DNA	SSAW	WFV(SSAW)
Protein	SSAW	SSAW
Simulated	SSAW(WFV)	SSAW

## Discussion

The proposed SSAW is inspired by the work WFV reported in [41]. In Bao et al.’s work [41], WFV was compared to five state-of-the-art methods, namely, *k*-tuple [4, 30], DMK [31], TSM [36], AMI [29] and CV [32] on DNA data set. WFV demonstrated overwhelming superiority over each of these methods. Because the proposed SSAW are better than WFV in clustering on each of the three types of data considered, we can expect that SSAW will have competitive (if not better) performance (with respect to both accuracy and speed) when compared against these five state-of-the-art methods. Classification performance was not examined in the original Bao et al.’s work [41].

Similarly, in [18], the $K_{2}^{*}$ method was compared to over 9 other alignment-free algorithms, especially, those that consider sequences in a pairwise manner (such as the general *D*_2_-family). The $K_{2}^{*}$ was shown to outperform most of the methods in this category. Thus, we expect that the relative performance of the proposed SSAW method over $K_{2}^{*}$ gives us an idea on how it will perform when compared with the *D*_2_-family, and other methods investigated in [18].

SSAW generally outperformed WFV with respect to accuracy, and the F-score measure. The performance improvement of SSAW over WFV can be attributed to two key factors: (1) the use of the stationary discrete wavelet transform which is able to keep information better during the transformation process than the standard discrete wavelet transform used in [41]; (2) The use of an improved representation for the *k*-mers, based on the initial mapping to complex numbers using the unit circle, before performing the wavelet transformation.

For clustering, SSAW outperformed $K_{2}^{*}$. This could be due to several reasons, for instance, the two points already mentioned above. Further, while $K_{2}^{*}$ needs to compare sequences pair by pair, SSAW and WFV do not need to compare two sequences in a pairwise manner. Rather, they generate a series of numbers to represent all sequences together which are then transformed into a feature vector. Hence, these two wavelet-based methods are more suitable for clustering than $K_{2}^{*}$.

Comparing WFV and SSAW in classification on DNA sequences, for short sequence (less than 1000 bp), SSAW produced better results. SSAW was slower on DNA classification which had relatively longer sequences (i.e, DNA data with an average sequence length of 1495 bp). It appears that SSAW is not suitable for long sequences, from a small alphabet. However, for larger alphabets, such as protein sequences (with an average sequence length of 497 bp), SSAW showed superior performance over both WFV and $K_{2}^{*}$.

SSAW did not perform well in generating the phylogenetic tree and in evaluating functionally related regulatory sequences. This is not too surprising, given the observed performance of WFV on these problems (see [18] for comparison with $K_{2}^{*}$).

The distance measurement used in SSAW is based on the simple Eucliean distance between two vectors. Luczak et al. [5] provided a recent comprehensive survey using different statistics to evaluate sequence similarity in alginment-free methods. After studying over 30 statistics (more than 10 basic measurements and their combinations), Luczak et al. [5] showed that simple single statistics are sufficient in alignment-free *k*-mer based similarity measurement. The Eucliean distance approach used in this work is thus just one approach to the distance measurement. Certainly, other distance measures, such as the earth mover distance, can be considered to further improve the proposed SSAW approach. Similarly, classification and clustering were performend using simple algorithms. Further improvement may be realized with more sophisticated analysis methods, e.g., using random forests for classification.

One of the main advantages of SSAW is the running time. SSAW is much faster than the other two methods, showing orders of magnitude improvement in execution time, while maintaining competitive (if not better) accuracy. Considering the huge volumes of data involved in most modern applications, and the rate at which these datasets are being generated, the rapid processing speed of alignment-free methods becomes a key factor. The proposed SSAW provides very rapid processing, without an undue loss in accuracy. This makes SSAW an attractive approach in most practical scenarios.

## Conclusions

A new alignment-free model for similarity assessment is proposed. We call it SSAW – Sequence Similarity Analysis using the Stationary Discrete Wavelet Transform. Three types of data are used in the study, DNA sequences, protein sequences, and simulated next-generation sequences. Two different applications, clustering and classification are considered. Compared with state-of-the-art methods, WFV, and *K*_2_∗, the proposed SSAW demonstrated competitive performance (accuracy, F-score, precision, and recall) both in clustering and classification. It also exhibited faster running times compared with the other methods. These make SSAW a practical approach to rapid sequence analysis, suitable for dealing with rapidly increasing volumes of sequence data required in most modern biological applications.

## Abbreviations

AMI: Average mutual information model which is proposed in paper [29]
CPU: Central processing unit
CV: A method which is proposed in paper [32]
CWT: Continuous wavelet transformation
DNA: Deoxyribonucleic acid
DMK: Distance measure based on *k*-tuples model which is proposed in paper [31]
DWT: Discrete wavelet transform
FFT: Fast fourier transformation
FN: False negative
FP: False positive
GHz: Giga-Hertz
GB: Gigabyte
MATLAB: A software package which is developed by Mathworks Inc, Natick, MA, USA, https://www.mathworks.com/
MRF: Markov Random Field (MRF)
PBIL: PBIL is abbreviation of PRABI-Lyon-Gerland. It is the protein database which is created in January 1998, which is located at the institute of Biology and Chemistry of Proteins IBCP ftp://pbil.univ-lyon1.fr/pub/hogenom/release_06/
RAM: Random access memory
SBARS: Spectral-based approach for repeats search method which is proposed in paper [42]
SSAW: Sequence Similarity Analysis method based on the stationary discrete Wavelet transform
SWT: Stationary wavelet transform
TN: True negative
TP: True positive
TSM: Three symbolic sequences model which is proposed in paper [36]
WFV: Wavelet-base feature vector model which is proposed in paper [41]
